# Development of an intervention for middle adolescents (15–17 years old) using the Behaviour Change Wheel (BCW) to empower adolescent decision-making regarding HPV vaccination uptake

**DOI:** 10.1371/journal.pone.0355229

**Published:** 2026-08-14

**Authors:** Terri Flood, Ciara M. Hughes, Iseult M. Wilson, Marian McLaughlin

**Affiliations:** 1 School of Health Sciences, Ulster University, Derry~Londonderry, United Kingdom; 2 School of Nursing and Midwifery, Queen's University Belfast, United Kingdom; 3 School of Psychology, Ulster University, Coleraine, United Kingdom; Kasturba Medical College Manipal, INDIA

## Abstract

**Introduction:**

In the United Kingdom, where a school-based human papillomavirus vaccination programme vaccinates 12–13 year old students, parents/guardians have been the primary decision-makers regarding receipt of the vaccination. As the average age of first sexual intercourse is 15–17 years old, education at this time could provide an opportunity for unvaccinated students to self-consent to vaccination. The aim of this study was to develop a HPV intervention, aimed at middle adolescents (15–17 years old), using the 3 stages of the Behaviour Change Wheel.

**Methods:**

A systematic review informed target behaviours and populations. Focus groups and interviews were conducted throughout Northern Ireland with immunisation nurses (n = 26), middle adolescents (n = 34), and post-primary school teachers (n = 12) and nurses (n = 6). Data analysis was guided by the COM-B model to inform a Behavioural Diagnosis. Subsequently, appropriate intervention functions, policy categories and Behaviour Change Techniques were selected with consideration of APEASE criteria.

**Results:**

The systematic review highlighted the lack of interventions designed for middle adolescents; existing interventions were sparse, commonly not based on behavioural theory and focused on female cancers. The study identified twelve barriers to three target behaviours which included: empowering students to make decisions about HPV; empowering professionals’ to design and deliver the intervention; and increasing positive public attitudes regarding HPV vaccination. A behaviour change HPV intervention was developed which included school-based HPV education aligned with mandated curriculum changes, alongside vaccination opportunity. The study identified content, mode of delivery and desirable qualities/characteristics of a professional who could design/deliver the HPV education. A public media campaign was deemed important in supporting this central education.

**Discussion and conclusion:**

This study highlights political, social and cultural barriers to HPV intervention success and the need for Public Health Authority and Education Authority support. A feasibility pilot study should be undertaken to assess the real-world practicality of this HPV intervention.

## Background

The human papillomavirus (HPV) is a sexually transmitted virus and is a strong aetiological factor in the development of cancers of the cervix, vulva, vagina, penis, anus and oropharynx [1–3]. Unlike other sexually transmitted infections (STIs), preventative vaccines can protect against HPV transmission and have been available since 2006 [4]. By November 2024, 144 of the 194 World Health Organisation (WHO) Member States had introduced national HPV vaccination programmes, with approximately 60% delivered through school-based programmes [4,5]. In the UK, a school-based programme was introduced for females aged 12–13 years in 2008 and extended to males of the same age in 2019 [6].

Prior to the COVID-19 pandemic, 82–85% of females received at least one HPV vaccine in the first year of post-primary school during the 2018–2019 academic year across England, Scotland, Wales and Northern Ireland (NI) [7–10]. However, since COVID-19, HPV vaccination uptake has declined significantly, with 2023–2024 data indicating that only 72–75% of females received at least one dose in the first year of post-primary school, while uptake among males was approximately 6% lower across all UK countries [11–14]. Similar fluctuations in HPV vaccination uptake have been observed internationally, often linked to media coverage and public discourse. For example, following widespread media attention questioning vaccine safety in Denmark in 2015, HPV vaccination uptake declined rapidly from approximately 90% to less than 50% of eligible girls receiving the first dose [15]. A comparable decline occurred in the Republic of Ireland, where an online campaign undermining HPV vaccination contributed to uptake falling from 87% in 2015 to 50% in 2016 [16], before recovering to over 78% by 2022–2023 [17].

Given the young age at which HPV vaccination is offered, parents and guardians have traditionally been the primary decision-makers regarding vaccine uptake [18,19]. Across the UK, parents receive HPV-related information through invitation letters and standardised National Health Service (NHS) information leaflets [20–23]. However, studies consistently demonstrate low parental knowledge of HPV, particularly in relation to male HPV-associated risk [24–27]. Some parents express concerns regarding vaccine safety or fear that vaccination may encourage earlier sexual activity, despite robust evidence demonstrating vaccine safety and no association between HPV vaccination and earlier sexual debut [28].

A systematic review by López et al. (2020) found that HPV knowledge among adolescents ranged from low to modest across multiple European countries [29]. Studies conducted outside Europe report similarly poor levels of HPV knowledge among adolescents [29–31]. Several studies further indicate that HPV knowledge among male adolescents is significantly lower than among females [18,30,31]. Socioeconomic deprivation has also been identified as a key determinant of lower HPV vaccination uptake and cervical screening participation, with the highest levels of non-participation observed in the most deprived populations [32–36].

The average age of first sexual intercourse in the UK and many other developed countries ranges from 15 to 18 years [37–43]. Providing HPV education to adolescents within this age group is therefore critical, as it coincides with increased sexual activity and growing autonomy in health-related decision-making. Such education may also facilitate opportunities for adolescents to self-consent to HPV vaccination. In many developed countries, including the UK, Canada, Sweden and parts of the US and Australia, adolescents under 16 years of age may legally consent to vaccination if deemed Gillick competent [44]. Gillick competence refers to a young person’s ability to understand the rationale for, and consequences of, a healthcare decision [44,45]. However, despite supportive legislation, school staff and healthcare professionals frequently report reluctance to facilitate adolescent self-consent within school settings due to concerns regarding parental opposition [44].

A recent systematic review, conducted by the authors and focusing on adolescents aged 15–17 years, found that while school-based HPV education can improve knowledge, evidence of sustained impact on vaccination uptake remains limited [46]. The review highlighted that most evaluated interventions target younger adolescents aged 11–13 years and are delivered at the point of vaccine offer, with relatively few evaluated interventions specifically targeting middle adolescents aged 15–17 years in school-based settings, who possess greater cognitive maturity and, in many settings, legal capacity to self-consent. Adolescents, educators and immunisation staff also identified multiple structural and motivational barriers to effective delivery of HPV education. Together, these findings indicate the need for a systematically developed, theory-informed intervention specifically tailored to adolescents aged 15–17 years, integrating education, decision-making and access to vaccination.

This study involved the recruitment of participants in Northern Ireland, United Kingdom. Northern Ireland represents a distinctive sociocultural context for HPV vaccination decision-making due to high levels of religious affiliation, faith-based school governance and historical school segregation, all of which have been shown to shape attitudes towards sexual health education [47–54]. Stigma surrounding STIs, coupled with parental, religious and community norms, has been identified as a barrier to open discussion of sexual health within schools, often resulting in cautious or inconsistent delivery of education [47–49,55,56]. These contextual factors may disproportionately limit adolescents’ access to accurate HPV information and opportunities for informed decision-making, particularly in settings where concerns about parental backlash or institutional ethos constrain educational practice [55–57]. Understanding this cultural and educational landscape is therefore essential when designing interventions intended to support adolescent autonomy and equitable access to HPV vaccination in Northern Ireland.

**Fig 1:**
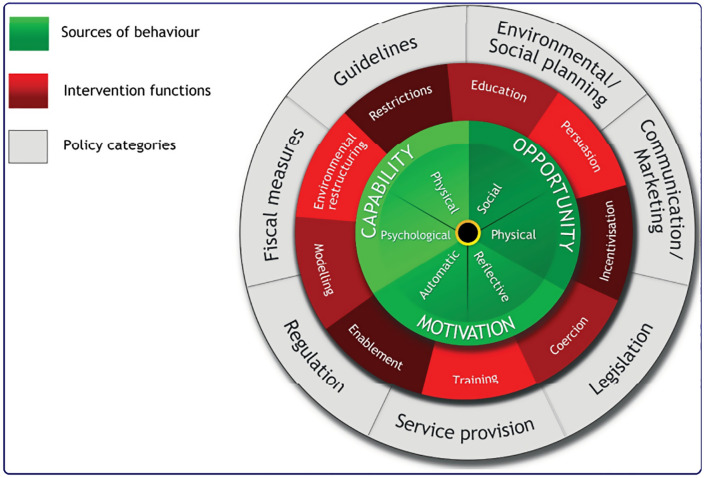
Behaviour change wheel [59].

In this study, the Behaviour Change Wheel (BCW), as described by Michie et al. [58,59], was applied to guide intervention development. The BCW was developed through systematic analysis of 19 behaviour change frameworks and provides a structured approach to intervention design based on three stages; 1) understanding the behaviour, 2) identifying mechanisms of change, and 3) selecting appropriate intervention strategies [59]. Central to the BCW is the COM-B model, which conceptualises behaviour as resulting from interactions between capability, opportunity and motivation [59]. The components of the BCW are outlined in Fig 1.

The BCW has been widely used to inform vaccination and sexual health interventions internationally. For example, Garbutt et al. [60] applied the framework to develop an intervention to increase HPV vaccination uptake in paediatric primary care settings in the United States, while Marshall et al. [61] developed a video-based behavioural intervention that improved HPV knowledge and vaccination intentions among parent–daughter dyads in Ireland. The BCW has also been successfully applied to sexual health and contraception interventions [62].

The aim of this study was to apply the Behaviour Change Wheel to synthesise findings from a systematic review and multiple qualitative studies in order to develop a theory-informed intervention to support informed HPV vaccination decision-making among adolescents aged 15–17 years.

## Methods

### Ethical approval

This study received ethical approval from the NHS Research Ethics Committee in September 2020 via the Integrated Research Application System (IRAS) (ID: 287358). Prior to submission, the protocol was reviewed by the Ulster University Institute of Nursing and Health Sciences Research Governance Filter Committee, which confirmed appropriate peer review and patient and public involvement. Following NHS approval, the project was approved by each of the five Health and Social Care Trusts in Northern Ireland.

### Study design

This study reports the development of a complex behavioural intervention to empower middle adolescents (15–17 years) to make informed decisions regarding HPV vaccination. The work represents the integrative synthesis stage of a wider programme of research, rather than a standalone qualitative analysis. The BCW framework was used to systematically synthesise evidence from a published systematic review and multiple qualitative studies to inform intervention design.

The purpose of this paper is therefore to demonstrate how evidence from multiple sources was mapped onto the BCW to progress through its three stages rather than to re-present detailed findings from each contributing study.

To develop a comprehensive understanding of the behavioural problem, evidence was drawn from four primary sources:

1. A systematic review examining the impact of school-based HPV educational interventions for adolescents aged 15–17 years [46].
2. Qualitative focus groups with school immunisation nurses [63].
3. Qualitative focus groups with students aged 15–16 years [64].
4. Qualitative interviews with post-primary school teachers and school-based nurses [65].

### Stage 1: Understanding the behaviour (behavioural diagnosis)

Stage 1 of the BCW focuses on developing a behavioural diagnosis by identifying what needs to change for the target behaviour to occur [58]. In this study, the behavioural diagnosis was informed through secondary synthesis of findings from the systematic review [46] and qualitative studies [63–65].

The COM-B model provided the overarching structure for behavioural diagnosis, conceptualising behaviour in terms of capability, opportunity and motivation [59]. In line with BCW guidance, the Theoretical Domains Framework (TDF) was specified to support the application of the COM-B model during the behavioural diagnosis stage, enabling categorisation of barriers and facilitators within each COM-B component [58,66]. The TDF identifies 14 theoretical domains associated with behaviour change [66].

In the original qualitative studies, data were analysed using directed content analysis with TDF domains used as predefined coding categories [66,67]. The only exception was the analysis of interviews with post-primary teachers and school-based nurses, where early findings indicated that participants did not perceive themselves as appropriate intervention designers or deliverers; consequently, thematic analysis was employed [65].

Coding across studies was conducted independently by at least two researchers, with discrepancies resolved through discussion to reach consensus. Formal inter-coder reliability statistics were not calculated, consistent with qualitative methodological guidance [67]. For the present study, published COM-B and TDF findings were synthesised rather than re-analysed as primary qualitative data.

This synthesis enabled identification of the key COM-B components requiring change across stakeholder groups (students, parents, nurses and teachers), forming a consolidated behavioural diagnosis to guide intervention development.

### Stage 2: Identification of intervention options

Stage 2 involved selecting intervention functions and supporting policy categories to address the behavioural diagnosis identified in Stage 1, following BCW guidance [58]. Intervention functions were selected from the nine BCW functions proposed by Michie et al. [58], informed by:

- The COM-B components identified in the behavioural diagnosis
- Evidence from the systematic review [46] and qualitative studies [63–65] regarding feasibility and acceptability
- Systematic application of the APEASE criteria

Within the BCW, the APEASE (Affordability, Practicality, Effectiveness and cost-effectiveness, Acceptability, Side-effects/safety and Equity) criteria are explicitly intended to guide the selection and justification of intervention functions and policy categories once a behavioural diagnosis has been established [58]. In this study, APEASE was applied as a structured decision-making tool to ensure that selected intervention options were not only theoretically appropriate but also implementable within real-world school and public health systems.

APEASE judgements were reached through researcher consensus, informed by empirical findings from the qualitative studies [63–65] and consideration of the Northern Ireland policy and service context. Supporting policy categories were then selected using the BCW policy framework [58], with emphasis placed on options judged most likely to support sustainable implementation without disproportionate cost or unintended consequences.

### Stage 3: Identification of intervention content and implementation options

Stage 3 focused on identifying Behaviour Change Techniques (BCTs) and modes of delivery to operationalise the selected intervention functions. A BCT is an ‘active component of an intervention designed to change behaviour’ (page 16) [58]. Any single intervention function may be delivered through a number of BCTs [46].

BCT selection was guided by:

- The behavioural diagnosis (COM-B/TDF)
- The intervention functions selected in Stage 2
- Behaviour Change Technique Taxonomy (Version 1) training [68]
- The BCT Theory and Techniques Tool [69]
- Continued application of the APEASE criteria [58]

BCTs were independently selected by two researchers trained in BCT taxonomy use [68], with agreement reached through discussion. Selected BCTs were mapped explicitly to COM-B components and target behaviours.

Modes of delivery were informed by evidence from the systematic review [46] and stakeholder preferences identified in the qualitative studies [63–65].

The multidisciplinary research team, comprising expertise in public health, psychology, education, nursing and behavioural science, supported reflexive interpretation of findings throughout the intervention development process. Reporting of qualitative components adhered to COREQ guidance [70], as detailed in the original qualitative publications [63–65].

Qualitative datasets are not publicly available due to confidentiality and ethical restrictions. However, detailed analytic procedures and findings are available in the cited primary publications [46,63–65], ensuring transparency and reproducibility of the behavioural diagnosis underpinning this intervention.

**Fig 2:**
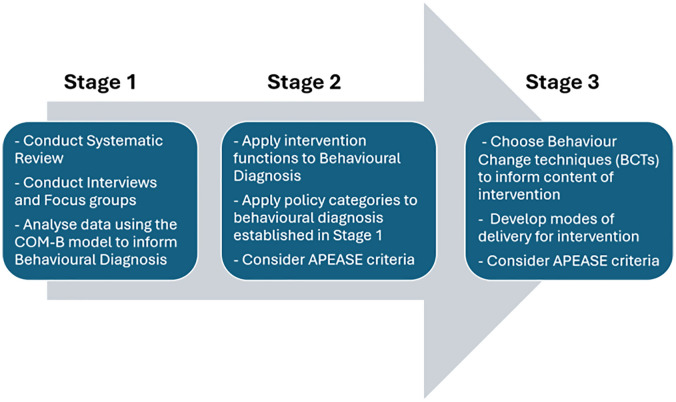
Application of the 3 Stages of the BCW to this study.

Fig 2 illustrates the application of the three stages of the Behaviour Change Wheel to the development of this intervention.

## Results

### Stage I: Understanding the behaviour

This stage involved exploring specific behavioural contexts of the problem, which included the social and environmental context that leads to poorer HPV knowledge, lower levels of HPV vaccination uptake and reduced involvement in HPV decision-making in middle adolescents in post-primary schools.

The researchers’ systematic review explored the impact of existing interventions on HPV vaccination uptake, HPV knowledge and impact on sexual behaviours [46]. Fifteen studies were included in the review which had explored HPV school-based interventions specifically including students 15–17 years old. Only five of these studies were found to include males and eight studies did not provide any details of theoretical models that they used to guide them in the planning and development of their educational intervention. Additionally, the content of the interventions largely focused on cervical cancer with only five of the fifteen studies including both genders.

Based on the systematic review, the researchers identified the need for the development of an age-specific intervention for this adolescent group, targeting certain behaviours which could impact the success of a HPV intervention. Criteria considered (as recommended by Michie et al. 2014 [58]), included the impact of each behaviour change and ability to measure behavioural change. Three behaviours were deemed important to target in order for the HPV intervention to have a positive and sustainable impact (see Table 1).

**Table 1:** Identified target behaviours of intervention.

	Target Behaviour
**1.**	Empowering middle adolescents to make decisions regarding HPV vaccination and reduce their risk of HPV acquisition
**2.**	Empowering suitable professionals to design and deliver a HPV intervention
**3.**	Increasing positive public attitudes towards HPV vaccination

Potential facilitators who play an important role in the design and delivery of a HPV intervention were identified most frequently as professionals with a nursing, healthcare or medical background [46]. Consequently, key stakeholders who could impact these specific behaviours were identified as: students aged 15–17 years; nurses associated with post-primary school education; and post-primary school teachers.

For pragmatic reasons, the structure of qualitative discussions varied by group. While face-to-face interviews are traditionally the ‘gold standard’ [71], both in-person and online formats yield high-quality data [72,73]. Due to COVID-19 restrictions in hospitals, online focus groups were used with Immunisation Nurses (IMNs) [63]. Face-to-face focus groups were held with students once restrictions eased to ensure a safe, comfortable environment [64]. For geographically dispersed post-primary school teachers and nurses, individual online interviews were deemed to be most suitable [65].

All participants provided written informed consent and parental consent was obtained from parents of the 15–17 year old students within the student focus groups. See Table 2 for full details of qualitative focus groups and interviews.

**Table 2:** Overview of participant groups.

Dates of interviews/focus groups	Participant group	Number of qualitative interviews/focus groups	Number of Participants
01 January 2021–01 June 2021	Immunisation Nurses (IMNs)	Six focus groups including IMNs from all Health and Social Care Trusts in NI.	26
05 November 2021–06 May 2022	Students 15–17 years old	Seven focus groups from four post-primary schools in NI.	34
01 February – 01 August 2022	Post-primary school teachers	Individual interviews with teachers from ten post-primary schools.	12
01 May 2022 – 01 August 2022	Post-primary school nurses	Individual interviews with nurses from six post-primary schools in NI.	6
**Total number of participants**			**78**

Across all studies, four COM-B components were identified as needing to change to permit the target behaviours (behavioural diagnosis): 1) Psychological Capability (Knowledge); 2) Physical Opportunity (Environmental Context & Resources); 3) Social Opportunity (Social Influences) and 4) Reflective Motivation (Beliefs about capabilities). While these domains were evident in all stakeholder groups, their manifestation varied according to role and context, with clear interdependencies between groups.

#### 1. Psychological capability (Knowledge).

Students demonstrated limited and fragmented knowledge of HPV, particularly regarding its status as a STI, risks to males, and the scope of protection offered by vaccination [64]. Most were unaware of their own vaccination status and reported minimal engagement with HPV-related education in school. In contrast, IMNs and school-based nurses reported high levels of HPV knowledge and confidence in vaccine safety and effectiveness [63,65]. However, they identified gaps in skills related to designing age-appropriate educational materials and delivering structured classroom-based education [63].

Teachers reported lower psychological capability than healthcare professionals, describing limited knowledge of HPV and low confidence in delivering HPV education, with most rejecting a role in intervention delivery beyond facilitation or gatekeeping [65]. Across groups, uncertainty regarding adolescents’ legal rights to self-consent for vaccination was evident, highlighting a shared knowledge gap with implications for adolescent autonomy [63–65].

#### 2. Physical opportunity (Environmental context and resources).

All stakeholder groups reported limited physical opportunity to support effective HPV education. Students described inconsistent or absent HPV content within relationship and sexuality education, often reduced to minimal references within science curricula [64]. Teachers corroborated this variability, attributing it to lack of mandated curriculum guidance, competing academic priorities and school ethos [56]. Nurses similarly highlighted the absence of protected time, resources and formal structures to support HPV education alongside vaccination delivery [63,65].

Despite these constraints, there was strong convergence across stakeholders that HPV education should be delivered within schools and paired with on-site access to vaccination for older adolescents [63–65]. This reflects an interrelationship between educational provision and service delivery, with opportunity for vaccination dependent on coordinated structural support.

#### 3. Social opportunity (Social influences).

Social opportunity barriers were consistently identified across groups, particularly in relation to parental influence, school culture and community norms. Students reported limited involvement in vaccination decisions, with parental consent processes restricting their autonomy [64]. Teachers and nurses described parental beliefs, religious values and fear of backlash as major influences on school engagement with HPV education and adolescent self-consent [63,65].

However, teachers differed from nurses in anticipating greater parental acceptance of HPV education for older adolescents, suggesting perceived variation in parental openness as adolescents mature [65]. Across groups, stigma surrounding sexual health and exposure to misinformation, particularly via social media, were identified as reinforcing social barriers to open discussion and informed decision-making [63–65].

#### 4. Reflective motivation (Beliefs about capabilities).

Students expressed motivation to protect themselves against HPV once personal risk was made salient, but often underestimated their susceptibility in the absence of vaccination [64]. Immunisation nurses and school-based nurses demonstrated strong professional motivation to design and deliver HPV education, viewing this as aligned with their role, though some reported reduced self-efficacy related to public speaking and classroom delivery [63,65].

Teachers’ reflective motivation was constrained by beliefs about role legitimacy and capability rather than opposition to HPV education itself, resulting in disengagement from delivery while still recognising its importance [65]. These differing motivational profiles highlight how stakeholder-specific beliefs interact to shape the feasibility of intervention delivery.

*Integrated behavioural diagnosis across stakeholder groups.* Taken together, findings indicate that barriers and facilitators to HPV education and vaccination operate across and between stakeholder groups, rather than within isolated roles. Students’ limited knowledge and autonomy [64] are reinforced by teachers’ reluctance to deliver HPV education [65] and by structural constraints identified by nurses [63]. Conversely, nurses’ motivation to lead HPV education is contingent on institutional support, time allocation and training [63,65].

This integrated behavioural diagnosis suggests the value of developing a multilevel intervention that simultaneously enhances psychological capability, restructures physical and social opportunity, and supports reflective motivation across students, educators and healthcare professionals.

*Contradictory and context-dependent stakeholder perspectives.* Contradictory views regarding HPV education content and format were reported across studies, particularly within the COM-B domains of social opportunity and reflective motivation [63–65]. Students and healthcare professionals supported comprehensive coverage of HPV transmission routes, including oral and anal sex, whereas some teachers expressed concern that discussing these topics could provoke parental backlash, particularly in faith-based schools [63,65].

Preferences regarding gender composition of HPV education sessions also varied. Most students, teachers and nurses favoured mixed-gender sessions [63,64], while a minority of teachers and female students, predominantly within single-sex Catholic schools, reported discomfort with mixed-gender discussions of sexual health [65]. Preferences regarding terminology differed by student gender, with male students favouring scientific language to maintain professionalism, and female students indicating that inclusion of non-scientific terminology enhanced understanding. Some stakeholders (from all stakeholder groups) anticipated disruptive behaviour among male students.

Table 3 provides a summary of the behavioural analysis and diagnosis of the target behaviour aligned to Michie et al.’s guidelines [1] mapping to the COM-B model and TDF domains.

**Table 3:** Summary of behaviour analysis and diagnosis of the target behaviours.

COM-B components	Theoretical domains framework	Action required	Target behaviour
***1. Psychological capability***	• Knowledge	1.1 Students need to be provided with reliable information about HPV vaccination and transmission.	1. Empowering middle adolescents to make decisions regarding HPV vaccination and reduce their risk of HPV acquisition
		1.2 Parents need to be educated about HPV.	3. Increasing positive public attitudes towards HPV vaccination
		1.3 Students, teachers, parents and nurses should understand the legal rights of students to consent to vaccination.	1. Empowering middle adolescents to make decisions regarding HPV vaccination and reduce their risk of HPV acquisition 2. Empowering suitable professionals to design and deliver a HPV intervention 3. Increasing positive public attitudes towards HPV vaccination
		1.4 Facilitators like specialist nurses, will require teacher and social media platform skills training to ensure high quality consistent delivery of a HPV intervention.	2. Empowering suitable professionals to design and deliver a HPV intervention
***2. Physical opportunity***	• Environmental context	2.1 Students need to have government mandated HPV education as part of a consistent spiral sexuality education curriculum, developing in complexity from the first year of post-primary school.	1. Empowering middle adolescents to make decisions regarding HPV vaccination and reduce their risk of HPV acquisition 2. Empowering suitable professionals to design and deliver a HPV intervention
		2.2 Students who are 16 years old should be provided with the opportunity to self-consent to the HPV vaccination.	1. Empowering middle adolescents to make decisions regarding HPV vaccination and reduce their risk of HPV acquisition
		2.3 Students who are 15 years old should be assessed for Gillick competence.	1. Empowering middle adolescents to make decisions regarding HPV vaccination and reduce their risk of HPV acquisition
	• Resources	2.4 The HPV knowledge and psychological skills of IMNs and school-based nurses needs to be acknowledged and utilised.	2. Empowering suitable professionals to design and deliver a HPV intervention
		2.5 Students need to have access to HPV vaccination at the time of the HPV intervention at 15–17 years old.	1. Empowering middle adolescents to make decisions regarding HPV vaccination and reduce their risk of HPV acquisition
		2.6 Time needs to be provided to facilitators to deliver HPV education alongside delivery of the vaccination.	2. Empowering suitable professionals to design and deliver a HPV intervention
***3. Social opportunity***	• Social Influences	3.1 Public campaigns are needed to highlight the potential impact of HPV, especially to males.	3. Increasing positive public attitudes towards HPV vaccination
***4. Reflective motivation***	• Beliefs about capabilities	4.1 Facilitators, like specialist nurses, will require confident-building exercises to increase their self-efficacy in terms of public speaking and presenting.	2. Empowering suitable professionals to design and deliver a HPV intervention

### Stage 2: Identification of intervention options

Once the behavioural analysis and diagnosis of the target behaviour were completed, the next stage involved identifying the functions of the intervention and supporting policy categories to achieve the desired behaviours. Michie et al. (2014) [58] proposes nine intervention functions and provides definitions and examples of each one, including the COM-B targets they are generally suitable to address. While West & Gould (2022) [74] indicate that the appropriate intervention functions to adopt will follow naturally from the behavioural diagnosis identified in Stage 1, APEASE criteria are also important to consider to determine the feasibility of all intervention functions [58]. Table 4 provides an overview of how the behaviour diagnosis and APEASE criteria (detailed in Methods section) informed the intervention functions selected. Exclusion decisions were informed by ethical and practical considerations specific to school-based delivery, including safeguarding, curriculum constraints, and proportionality for a preventative educational intervention. Consequently, five of the intervention functions aligned to the behavioural analysis and diagnosis of the target behaviour including: Education; Environmental Restructuring; Enablement; Modelling; and Training.

**Table 4:** APEASE Criteria linked to intervention functions.

BCW intervention functions	Definition of BCW intervention functions	COM-B targets from behavioural diagnosis linked to intervention functions	Does the intervention function meet the APEASE criteria?	Target behaviour
**Education**	Increasing knowledge and understanding	• Psychological capability • Reflective motivation	Yes – supports informed, voluntary decision-making without coercion	1,2,3
**Persuasion**	Using communication to induce positive or negative feelings or stimulate action	• Reflective motivation	No – not acceptable, could undermine informed consent and adolescent autonomy	None
**Incentivisation**	Creating an expectation of reward	• Reflective motivation	No – not acceptable, affordable or cost-effective in school-based setting for preventative vaccine	None
**Coercion**	Creating an expectation of punishment or cost	• Reflective motivation	No – not acceptable, violation of adolescent autonomy and legal rights	None
**Training**	Imparting skills	• Psychological capability • Physical opportunity	Yes – equips adolescents with skills	1,2
**Restriction**	Using rules to limit or direct opportunities for behaviours	• Physical opportunity • Social opportunity	No – not acceptable, limits adolescents’ ability to make an informed, voluntary decision about vaccination	None
**Environmental restructuring**	Changing the physical or social context	• Physical opportunity • Social opportunity	Yes – facilitates access and creation of support	1,2,3
**Modelling**	Providing an example for people to aspire to or imitate	• Social opportunity	Yes – informs and inspires choices	1,3
**Enablement**	Increasing means/reducing barriers to increase capability (beyond education and training) or opportunity (beyond environmental restructuring)	• Psychological capability • Physical opportunity • Social opportunity	Yes – enhances access while preserving adolescent informed decision-making	3

Michie at al. (2014) [58] provide a list of 7 policy categories in the BCW, including labels, definitions and examples as well as a matrix of links between intervention functions and policy categories. Table 5 demonstrates the policy categories linked to the chosen intervention functions for the intervention. The APEASE criteria is again utilised to establish the feasibility of each policy function. The 5 supporting policies deemed likely to be effective in bringing about change are: Service provision; Guidelines; Regulation; Environmental and Communications and Marketing.

**Table 5:** APEASE Criteria linked to policy categories.

Policy Category	Definition	Policy Category linked to chosen Intervention function	Does the policy category meet the APEASE criteria?
**Communication/ marketing**	Using print, electronic, telephonic or broadcast media	• Education • Modelling	Yes
**Guidelines**	Creating documents that recommend or mandate practice. This includes all changes to service provision	• Education • Training • Environmental restructuring • Enablement	Yes
**Fiscal measures**	Using the tax system to reduce or increase the financial cost	•Training • Environmental restructuring • Enablement	No – not affordable, practicable, cost effective or acceptable
**Regulation**	Establishing rules or principles of behaviour or practice	• Education • Training • Environmental restructuring • Enablement	Yes
**Legislation**	Making or changing laws	• Education • Training • Environmental restructuring • Enablement	No – not acceptable, legal protection established for adolescents
**Environmental/social planning**	Designing and/or controlling the physical or social environment	• Environmental restructuring • Enablement	Yes
**Service provision**	Delivering a service	• Education • Training • Modelling • Enablement	Yes

**Fig 3:**
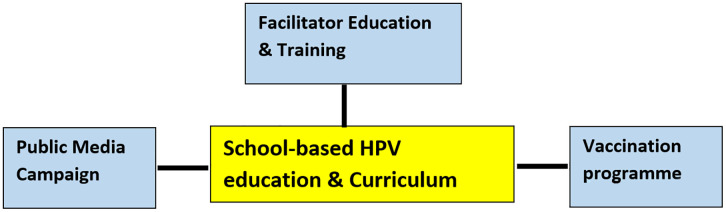
Visual representation of Proposed Intervention.

Through the process of identification of intervention functions and policy categories, a school-based HPV education supported by mandated curriculum changes, was determined to be the central component of the intervention alongside three additional intervention components; a Vaccination programme; Facilitator Education & Training; and a Public Media Campaign (see Fig 3)

The following considerations were explored in terms of evaluating this intervention with the APEASE criteria:

*Affordability:* It is estimated that ten permanent part-time facilitators (0.2FT workload each) could work in teams of two to facilitate HPV education classes throughout the five trusts in NI. This was determined to be low cost in terms of human resources required. Resources already exist to develop the HPV education and facilitator training at little extra cost.

*Practicality:* Existing staff in NI have the skills and knowledge to facilitate the HPV education. The HPV education could be incorporated into the school Personal Development Programme. The HPV vaccination could be administered with other standard vaccinations administered at 15–17 years old. The PHA already has a public information campaigns function within its structure and have teams with the skills to develop this type of campaign [75].

*Effectiveness & Equality:* HPV education has the potential to increase acceptability of the HPV vaccination, providing all students, irrelevant of their background, the opportunity to make their own decisions about HPV and HPV vaccination.

*Cost-effectiveness:* This initiative has the potential to increase safer sexual behaviours and increase uptake of the HPV vaccine resulting in fewer health conditions, reducing burden on NHS services and related mortality.

*Side-effects/safety*: The HPV vaccine is a safe vaccination which is used globally, resulting in only limited mild side-effects.

Tables 6–8 provide intervention functions and policies linked to each target behaviour and intervention component.

**Table 6:** COM-B behavioural diagnosis for Target behaviour 1 linked to intervention functions and BCW Policy Categories.

TARGET BEHAVIOUR 1: Empowering middle adolescents to make decisions regarding HPV vaccination and reduce their risk of HPV acquisition					
Source of behaviour (COM-B)	Theoretical Domains Framework (TDF)	Intervention function	Mode of Delivery (Action required*)	Policy category	Behavioural Change Technique (BCT) Cluster (BCT Taxonomy)**
1. Psychological Capability	• Knowledge	• Education • Training	• School-based HPV education & Curriculum Changes (1.1,1.3)	• Service provision • Guidelines • Regulation	4. Shaping Knowledge (4.1–4.4) 5. Natural Consequences (5.1–5.3,5.6) 9. Comparison of Outcomes (9.1–9.3)
2. Physical Opportunity	• Environmental context • Resources	• Environmental restructuring • Enablement • Training	• School-based HPV education & Curriculum Changes (2.1-2.3) • Vaccination programme (2.5)	• Service provision • Guidelines • Regulation • Environmental/Social planning	12. Antecedents (12.5)
3. Social Opportunity	• Social influences	• Modelling • Environmental restructuring	• School-based HPV education & Curriculum Changes	• Service provision • Regulation • Guidelines • Environmental/Social planning	5. Natural Consequences (5.1–5.3,5.6)

* Action required as identified in Table 3

** BCT Clusters and Taxonomy as defined in Table 9

**Table 7:** COM-B behavioural diagnosis for Target behaviour 2 linked to intervention functions and BCW Policy Categories.

TARGET BEHAVIOUR 2: Empowering suitable professionals to design and deliver a HPV intervention					
Source of behaviour (COM-B)	Theoretical Domains Framework (TDF)	Intervention function	Mode of Delivery (Action required)	Policy category	Behavioural Change Technique (BCT) Cluster (BCT Taxonomy)**
1. Psychological Capability	• Knowledge	• Education • Training	• Facilitator education & training (1.3,1.4)	• Service provision • Guidelines	4. Shaping Knowledge (4.1)
2. Physical Opportunity	• Environmental context • Resources	• Environmental restructuring • Training	• School-based HPV education & • Curriculum Changes (2.1,2.4,2.6)	• Service provision • Guidelines • Regulation • Environmental	12. Antecedents (12.5)
4. Reflective Motivation	• Beliefs about capabilities	• Education	• School-based HPV education (4.1)	• Service provision • Guidelines	15. Self-belief (15.1–15.4)

* Action required as identified in Table 3

** BCT Clusters and Taxonomy as defined in Table 9

**Table 8:** COM-B behavioural diagnosis for Target behaviour 3 linked to intervention functions and BCW Policy Categories.

TARGET BEHAVIOUR 3: Increasing positive public attitudes towards HPV vaccination					
Source of behaviour (COM-B)	Theoretical Domains Framework (TDF)	Intervention function	Mode of Delivery (Action required)	Policy category	Behavioural Change Technique (BCT) Cluster (BCT Taxonomy)**
1. Psychological Capability	• Knowledge	• Education • Enablement	• School-based HPV education & Curriculum Changes (1.2,1.3) • Public Media Campaign (1.2)	• Service provision • Guidelines • Regulation • Communication/marketing	1. Goals and Planning (1.1,1.7)
3. Social Opportunity	• Social Influences	• Environmental restructuring • Modelling • Enablement	• Public Media Campaign (3.1)	• Communication/marketing	7. Associations (7.1) 12. Antecedents (12.2)

* Action required as identified in Table 3

** BCT Clusters and Taxonomy as defined in Table 9

### Stage 3: Identification of content and implementation options

This stage included the identification of BCTs and implementation options. The Behaviour Change Technique Taxonomy (BCTT) produced by Mitchie et al. (2013) provides 93 distinct, hierarchically ordered BCT’s with labels and definitions, divided into 16 clusters [76]. As in stage 2, these steps were informed through a review of previous research, conducting a systematic review and discussions with stakeholders in interviews and focus groups. Additionally, the intervention functions identified in stage 2 are also important in determining the BCTs. BCTs were selected independently by two researchers as described in the methods section. Subsequently, 20 out of the 93 BCTs from 7 BCT clusters, were considered appropriate to this intervention design (see Table 9 for additional details):

**Table 9:** The Selected BCTs with descriptions.

BCT Cluster	BCT Taxonomy	BCT Label	Practical Example	Target Behaviour
1. Goals and Planning	1.1 Goal setting	Set or agree on a goal defined in terms of the behaviour to be achieved	PHA to set HPV vaccination uptake goals and goals regarding intention to attend HPV screening	3. Increasing positive public attitudes towards HPV vaccination
	1.7 Review outcome goal	Review outcome goal(s) jointly with the person and consider modifying goal(s) in light of achievement	PHA to review achievement of goals regarding HPV vaccination uptake and intention to attend HPV screening	3. Increasing positive public attitudes towards HPV vaccination
4. Shaping Knowledge	4.1 Instruction on how to perform a behaviour	Advise or agree on how to perform the behaviour	Explain what students need to do in order to be vaccinated Instruct professional facilitators on how to present effectively	1. Empowering middle adolescents to make decisions regarding HPV vaccination and reduce their risk of HPV acquisition 2. Empowering suitable professionals to design and deliver a HPV intervention
	4.2 Information about antecedents	Provide information about antecedents (e.g., social and environmental situations and events, emotions, cognitions) that reliably predict performance of the behaviour	Educate students about social factors which can influence safe or unsafe sexual practices	1. Empowering middle adolescents to make decisions regarding HPV vaccination and reduce their risk of HPV acquisition
	4.3 Re-attribution	Elicit perceived causes of behaviour and suggest alternative explanations (e.g., external or internal and stable or unstable)	Educate students about personal factors which can influence safe or unsafe sexual practices	1. Empowering middle adolescents to make decisions regarding HPV vaccination and reduce their risk of HPV acquisition
	4.4 Behavioural experiments	Advise on how to identify and test hypotheses about the behaviour, its causes and consequences, by collecting and interpreting data	Educate students about reliable resources and how to identify misinformation.	1. Empowering middle adolescents to make decisions regarding HPV vaccination and reduce their risk of HPV acquisition
5. Natural Consequences	5.1 Information about health consequences	Provide information (e.g., written, verbal, visual) about health consequences of performing the behaviour	Explain to students that not getting the HPV vaccination increases risk of transmission of the virus	1. Empowering middle adolescents to make decisions regarding HPV vaccination and reduce their risk of HPV acquisition
	5.2 Salience of consequences	Use methods specifically designed to emphasise the consequences of performing the behaviour with the aim of making them more memorable	Use a variety of interactive teaching tools to increase the impact of HPV education	1. Empowering middle adolescents to make decisions regarding HPV vaccination and reduce their risk of HPV acquisition
	5.3 Information about social and environmental consequences	Provide information (e.g., written, verbal, visual) about social and environmental consequences of performing the behavior	Explain the risk of various types of cancer and genital warts from transmission of HPV	1. Empowering middle adolescents to make decisions regarding HPV vaccination and reduce their risk of HPV acquisition
	5.6 Information about emotional consequences	Provide an observable sample of the performance of the behaviour, directly in person or indirectly, e.g., via film, pictures, for the person to aspire to or imitate	Use video of celebrity or influencer with history of HPV to increase the impact of the education	1. Empowering middle adolescents to make decisions regarding HPV vaccination and reduce their risk of HPV acquisition
7. Associations	7.1 Prompts/Cues	Introduce or define environmental or social stimulus with the purpose of prompting or cueing the behavior.	Post posters in specific healthcare environments to reinforce public campaign message	3. Increasing positive public attitudes towards HPV vaccination
9. Comparison of Outcomes	9.1 Credible source	Present verbal or visual communication from a credible source in favour of or against the behavior	Ensure that the person delivering the student education has expertise and is respected by the students	1. Empowering middle adolescents to make decisions regarding HPV vaccination and reduce their risk of HPV acquisition
	9.2 Pros and cons	Advise the person to identify and compare reasons for wanting (pros) and not wanting (cons) to change the behaviour	Encourage discussion and reflection regarding the pros and cons of HPV vaccination	1. Empowering middle adolescents to make decisions regarding HPV vaccination and reduce their risk of HPV acquisition
	9.3 Comparative imagining of future outcomes	Prompt the person to imagine and compare likely or possible outcomes following attending versus not attending a screening appointment	Encourage discussion and reflection regarding the pros and cons of attending HPV screening	1. Empowering middle adolescents to make decisions regarding HPV vaccination and reduce their risk of HPV acquisition
12. Antecedents	12.2 Restructuring the social environment	Change, or advise to change the social environment in order to facilitate performance of the wanted behaviour or create barriers to the unwanted behaviour	Restructure students into groups to encourage discussion Encourage the PHA to promote open communication about sexual health and HPV	1. Empowering middle adolescents to make decisions regarding HPV vaccination and reduce their risk of HPV acquisition 3. Increasing positive public attitudes towards HPV vaccination
	12.5 Adding objects to the environment	Add objects to the environment in order to facilitate performance of the behaviour	Provide free condoms to facilitate safe sex Providing resources for facilitators to utilise to design and develop their HPV education	1. Empowering middle adolescents to make decisions regarding HPV vaccination and reduce their risk of HPV acquisition 2. Empowering suitable professionals to design and deliver a HPV intervention
15. Self-belief	15.1 Verbal persuasion about capability	Tell the person that they can successfully perform the wanted behaviour, arguing against self-doubts and asserting that they can and will succeed	Encourage specialist nurses to showcase their knowledge and skills to design and deliver HPV education	2. Empowering suitable professionals to design and deliver a HPV intervention
	15.2 Mental rehearsal of successful performance	Advise to practise imagining performing the behaviour successfully in relevant contexts	Highlight the benefits of practicing presentation skills in a safe environment prior to facilitating student sessions	2. Empowering suitable professionals to design and deliver a HPV intervention
	15.3 Focus on past success	Advise to think about or list previous successes in performing the behaviour (or parts of it)	Encourage nurses to reflect on situations where they were able to help students with HPV and/or sexual health related issues	2. Empowering suitable professionals to design and deliver a HPV intervention
	15.4 Self-talk	Prompt positive self-talk (aloud or silently) before and during the behaviour	Highlight the benefits of the HPV education to students and how much of a difference they can make to the student’s wellbeing	2. Empowering suitable professionals to design and deliver a HPV intervention

#### 1. Goals and planning.

Stakeholders consistently emphasised that successful implementation of any HPV intervention for middle adolescents would require leadership and strategic planning by the Public Health Agency (PHA). This included setting explicit goals to increase HPV vaccination uptake and to support future engagement with HPV screening programmes. Stakeholders identified mandating HPV education and providing vaccination opportunities for 15–17 year olds as key mechanisms for increasing physical opportunity, requiring coordination between the PHA and the Education Authority (EA), which holds responsibility for education delivery across Northern Ireland [77].

#### 4. Shaping knowledge.

Stakeholders supported mandating sexual health education, including HPV education, within post-primary schools as part of a spiral curriculum. Face-to-face, school-based HPV education was identified as the central intervention component to improve adolescent knowledge. Stakeholders indicated that small group delivery (6–10 students) was preferable for engagement, although classroom-sized groups (20–30 students) were considered acceptable where logistical constraints existed.

Students reported that receiving education alongside familiar peers fostered a sense of safety and openness, despite prior research suggesting that familiarity may increase peer pressure and under-disclosure [78]. Estimates of optimal session length ranged from 30 to 120 minutes; however, evidence from the accompanying systematic review indicated that interventions of shorter duration may also be effective [46].

Most stakeholders recommended reinforcing face-to-face education with dedicated social media content embedded within commonly used platforms such as Instagram or TikTok. Clear, transparent guidance regarding adolescents’ legal right to self-consent for vaccination at age 16 years and younger was identified as essential to support consistent understanding among students, teachers, and nurses. Stakeholders also reported poor parental knowledge of HPV.

#### 5. Natural consequences.

Stakeholders indicated that interactive teaching methods (e.g., quizzes, magazines, practical demonstrations, and question-and-answer sessions) should be incorporated to enhance engagement and retention of information related to the natural consequences of HPV acquisition. The use of high-profile public narratives, including reference to Jade Goody [79], was frequently highlighted.

Most stakeholders supported comprehensive coverage of HPV transmission, prevention, and vaccination. While vaginal sex was widely accepted as appropriate for discussion, some teachers expressed concern that addressing oral and anal transmission could provoke negative parental reactions. Other stakeholders emphasised the importance of inclusive discussion of all transmission routes, including same-sex relationships, noting limited diversity in existing educational practice.

#### 7. Associations.

Stakeholders identified the use of visual prompts as a mechanism to cue HPV-related behaviours. Suggested strategies included displaying HPV vaccination and screening posters within healthcare environments such as general practices and pharmacies to increase salience and encourage uptake.

#### 9. Comparison of outcomes.

Most teachers reported limited knowledge and confidence in HPV education and low motivation to deliver sexual health content, with the exception of a small number of science teachers. In contrast, students consistently reported greater attention and engagement when education was delivered by an external facilitator perceived as credible and knowledgeable.

There was strong agreement across stakeholder groups that HPV education should be delivered by a healthcare professional, with immunisation nurses viewed as particularly appropriate facilitators. While professional expertise was valued, facilitator openness and a non-judgemental approach were identified as the most important characteristics. Facilitator age was considered less influential, although some stakeholders suggested that younger facilitators might enhance engagement. Students also reported that discussing HPV with an external facilitator reduced embarrassment.

School-based nurses reported extensive training and experience in adolescent sexual health, including support for STIs and pregnancy. Despite this expertise often being under-recognised and limited availability across schools, school-based nurses expressed strong motivation to contribute to HPV education.

#### 12. Antecedents.

Stakeholders emphasised the stigma surrounding STIs within the sociocultural context of Northern Ireland. Addressing this was viewed as requiring a coordinated public media campaign led by the PHA, balancing risk-reduction messaging with sex-positive communication. In addition, stakeholders suggested that facilitators could provide sexual health resources, such as condoms and dental dams, during education sessions to support safer sexual practices.

#### 15. Self-belief.

National Minimum Standards and the Core Curriculum for Immunisation Training in the UK prepare immunisation nurses for HPV vaccine delivery and education [80]. Most immunisation nurses reported confidence in their qualifications and viewed HPV education as aligned with their professional role. However, some identified a need for additional training in pedagogical skills, including presentation delivery and engagement with social media platforms. Ensuring facilitators possess both subject-matter expertise and teaching competence was viewed as essential for delivering a consistent and high-quality educational experience [81].

## Discussion

This study applied the Behaviour Change Wheel (BCW) to design a theory-informed intervention aimed at empowering middle adolescents to make informed decisions regarding HPV vaccination and reducing their risk of HPV acquisition. A key strength of this work lies in the organised and transparent application of BCW guidance to intervention development [58,59,76]. To our knowledge, few HPV educational interventions grounded explicitly in behaviour change theory have been developed specifically for middle adolescents aged 15–17 years within school-based settings, a group who are increasingly autonomous and legally able to self-consent to vaccination in many countries, including the UK [46].

Following the BCW process, the central component of the intervention was identified as face-to-face, comprehensive, developmentally appropriate, school-based HPV education, supported by an ‘attention-grabbing’ social media platform. This education would be optimally embedded within a spiral sexuality curriculum and supported at policy level through public health and educational governance structures. Importantly, the curriculum should extend beyond abstinence-only approaches and, for middle adolescents, include discussion of HPV transmission routes, safer sex practices, and diverse sexual behaviours and identities. These recommendations closely align with Astle et al.’s 2021 study of college students’ suggestions for improving sex education in schools [82]. Consistent with stakeholder input, HPV education should incorporate multiple interactive teaching tools and be delivered by an open, confident healthcare professional with appropriate subject-specific expertise and pedagogical skills.

### Psychological capability and adolescent knowledge

Consistent with previous literature [29–31], findings from this study indicate that students in post-primary education in Northern Ireland have poor knowledge of HPV, with most unaware that HPV is an STI [64]. While some awareness of the link between HPV and cervical cancer was evident, knowledge of male HPV-associated cancer risk was notably absent, reflecting persistent gendered misconceptions reported elsewhere [57]. Limited recall of meaningful discussions with teachers or healthcare professionals further compounded these gaps, leaving many students unaware of their vaccination status until participation in the study.

Despite limited baseline knowledge, stakeholders widely agreed that adolescents aged 15–16 years possess sufficient maturity to engage with comprehensive HPV education and to self-consent to vaccination. However, uncertainty surrounding consent legislation and Gillick competence across all stakeholder groups highlights a shared capability gap that may undermine adolescents’ legal rights in practice; a concern raised in recent studies [83,84]. These findings reinforce the importance of developmentally appropriate, staged education that progressively builds knowledge and decision-making capacity across adolescence.

While the intervention proposed in this study seeks to respect adolescents’ decision-making capacity and legal rights to self-consent, stakeholders consistently emphasised the importance of supporting open communication between parents and adolescents. The provision of accessible, evidence-based parental educational resources was therefore viewed as an essential adjunct to adolescent-focused education.

### Physical opportunity and educational delivery

The findings highlight substantial deficits in physical opportunity for HPV education within post-primary schools. HPV content was frequently described as absent or minimal, often limited to a single reference in science textbooks, reflecting broader inconsistencies in sexual health education provision. This aligns with long-standing evidence supporting comprehensive sex education delivered throughout post-primary education and prior to sexual debut [85].

Stakeholders strongly supported mandating sexual health education, including HPV, within a spiral curriculum to ensure consistency and equity across schools. Importantly, HPV education should be paired with on-site access to vaccination, reinforcing evidence that co-locating education and service delivery enhances vaccine uptake. Successful implementation was viewed as contingent on the allocation of dedicated time and resources for specialist healthcare providers, underscoring the importance of environmental restructuring alongside educational content.

### Social opportunity, parental influence, and cultural context

Consistent with the behavioural diagnosis, parental and institutional influences emerged as dominant social opportunity constraints shaping adolescent HPV vaccination decision-making. Parental beliefs, culture, religion, and school ethos were repeatedly identified as barriers within this study and across existing literature [47–49,63–65]. Poor parental knowledge of HPV [24–27], particularly regarding male HPV-associated cancer risk [57], and misconceptions regarding the appropriateness of vaccination prior to sexual debut [55] are viewed as key drivers of delayed or withheld consent.

Teachers perceived that parents may be more receptive to HPV education and vaccination for adolescents aged 15–16 years, a view supported by evidence demonstrating increasing parental acceptance as adolescents age [86]. Kornides et al. [86] reported that 45% of parents who initially declined HPV vaccination later accepted it, with increasing adolescent age, improved parental knowledge, and provider recommendation identified as key influences. These findings support the importance of offering vaccination opportunities shortly after education delivery.

Education has been shown to increase parental support for vaccination [25,57], reinforcing the need for HPV interventions to incorporate targeted parental components. Differentiated approaches for passive versus active refusal, as proposed by Fisher et al. [87], may be particularly effective, with reminder-based strategies supporting passive refusers [88] and more intensive, person-delivered interventions required for active refusal [89].

The religious and cultural context of Northern Ireland introduces additional complexity. High levels of religious affiliation and school segregation [50–54] contribute to stigma surrounding sexual health, with teachers expressing concern about parental backlash when addressing sensitive topics. Students, however, strongly advocated for equitable and uncensored education regardless of religious affiliation. Supporting faith-based institutions to engage constructively with HPV education may therefore represent an important avenue for facilitating open dialogue while respecting religious values.

### Media influence and misinformation

Social media was widely recognised as a major source of HPV-related information and misinformation. Adolescents’ limited ability to distinguish credible from false information [90], alongside declining trust in traditional news sources [91], highlights the importance of integrating digital literacy and evidence-based messaging within HPV interventions. While social media interventions alone have demonstrated limited impact on vaccination uptake [92], they may play a valuable reinforcing role when combined with face-to-face education. First-person narratives and real-life stories, delivered via video or social media, were viewed as particularly powerful in increasing awareness of HPV-associated cancers, consistent with other studies [93–96].

Public media campaigns were identified as an essential intervention component. The Danish ‘Stop HPV – stop cervical cancer’ campaign demonstrated that personal narratives generated greater engagement and more positive dialogue than purely factual content [97,98], with subsequent increases in HPV vaccination rates [99]. Similar successes have been reported in the United States [100,101] and Italy [102]. Collectively, this evidence supports the use of combined narrative and factual approaches in public media campaigns, with UK initiatives needing to explicitly address both male and female HPV-associated cancers to correct persistent gendered misconceptions.

### Reflective motivation, stigma, and inclusivity

Students reported increased motivation to protect themselves against HPV when personal relevance was emphasised, particularly through family experiences and media narratives. However, underestimation of personal risk and limited understanding of vaccine scope remained barriers to sustained motivation.

Stigma surrounding STIs was pervasive across stakeholder groups, reinforcing the need for sex-positive, destigmatising communication strategies. Mass media campaigns have demonstrated effectiveness in improving sexual health knowledge and influencing behaviour, with greater exposure associated with greater impact [103–105]. Environmental cues, such as posters and digital prompts, may further reinforce messaging [106–109].

While all stakeholder groups supported comprehensive HPV education, differences emerged regarding content and delivery format. Some teachers expressed concern about discussing oral and anal transmission due to anticipated parental backlash, whereas other stakeholders viewed comprehensive coverage as essential. Inclusive curricula addressing diverse sexual orientations and identities have been shown to improve safety and reduce homophobia [110]. Differences in preferences for scientific versus non-scientific terminology reflected gendered interpretations of sexual language [111,112], highlighting the need for skilled facilitation to ensure sensitive and inclusive delivery [113].

### Facilitator role and intervention delivery

Across stakeholder groups, students consistently reported greater engagement when HPV education was delivered by an external healthcare professional, a finding supported by previous studies [114,115]. Immunisation nurses were viewed as particularly well placed to deliver HPV education, aligning with evidence that healthcare provider recommendation is among the strongest predictors of HPV vaccine uptake [116].

While school-based nurses demonstrated high levels of sexual health expertise and motivation, their role was frequently undervalued and access inconsistent across schools. Some immunisation nurses identified training needs related to pedagogical skills and social media engagement, reinforcing the importance of equipping facilitators with both subject-matter expertise and educational competencies [81].

### Implications for intervention design

Overall, the findings indicate the potential value of a multilevel intervention targeting individual, community, and policy determinants of HPV vaccination behaviour, consistent with socio-ecological and behavioural theory [117,118]. By addressing capability, opportunity, and motivation across students, parents, educators, and healthcare professionals, the proposed intervention offers a theoretically robust and contextually sensitive approach to improving HPV vaccination decision-making among middle adolescents.

**Fig 4:**
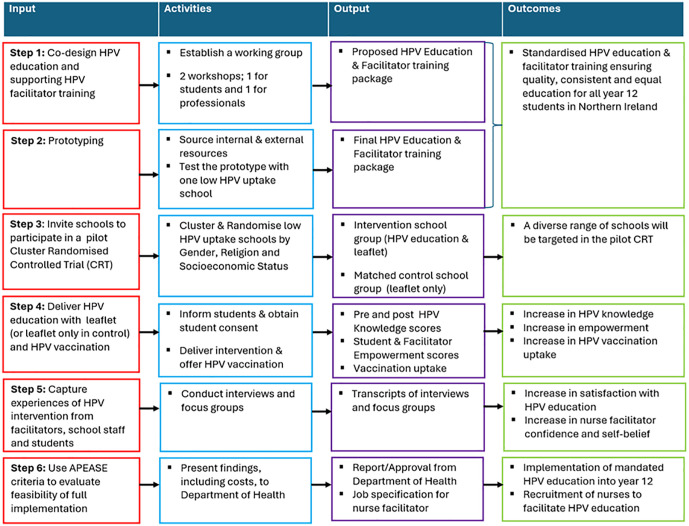
Proposed logic model for Pilot feasibility study.

To assess feasibility, cost, and implementation practicality, a person-centred pilot cluster randomised feasibility trial is warranted. Such a study would allow examination of acceptability, recruitment, retention, and potential sources of bias, and may inform refinement of intervention components prior to full-scale evaluation [119]. Fig 4 presents a logic model to support the proposed pilot feasibility study in Northern Ireland.

### Strengths and limitations

The key strength of this study lies in the structured and rigorous approach to designing behaviour change interventions through the BCW. The COREQ (COnsolidated criteria for REporting Qualitative research) Checklist [120] was used to ensure methodological rigour during the interview and focus group process, e.g., consistent interview and focus group guides; experienced and qualified interviewers; validation of coding by multiple researchers. Reflexivity was addressed through creation of an open environment and the presence of a second researcher observing the sessions. The qualitative sessions included representation from a range of highly skilled professionals.

This study has notable limitations. First, the intervention was developed through secondary synthesis of a systematic review and previously published qualitative studies rather than new primary data collection. While this approach is consistent with Behaviour Change Wheel guidance, it relies on the scope and analytic depth of the original studies [46,63–65] and does not include direct input from parents or policymakers.

Second, although multiple stakeholder groups were represented, the number of school-based nurses was limited, reflecting workforce availability in Northern Ireland, and data saturation for this subgroup cannot be confirmed. Third, the findings are context-specific to the educational, cultural and policy environment of Northern Ireland, and adaptation would be required for implementation in other settings.

## Conclusion

This study applied the Behaviour Change Wheel to synthesise evidence from a systematic review and multiple qualitative studies to develop an intervention supporting HPV vaccination decision-making among adolescents aged 15–17 years. By integrating perspectives from students, teachers, and nurses, the study identified interdependent determinants across psychological capability, opportunity, and motivation operating at individual, institutional, and social levels.

The key novelty of this work lies in its focus on middle adolescents, a group with increasing autonomy and, in many settings, legal capacity to self-consent to vaccination, for whom limited evaluated school-based, theory-informed HPV educational interventions were identified within the scope of our review. Unlike many earlier interventions designed primarily for younger adolescents at the point of vaccine offer, this intervention is developmentally tailored and explicitly informed by behaviour change theory using the Behaviour Change Wheel framework**.**

By transparently mapping empirical evidence to the COM-B model, this study provides a robust foundation for a multilevel, school-based intervention supported at policy level. While empirical testing is now required, the findings offer a strong platform for advancing adolescent engagement in HPV vaccination decision-making and informing intervention design in comparable educational and sociocultural contexts.
